# Mechanism Study of Acupuncture and Acupuncture Combined With Medication in the Treatment of Ulcerative Colitis

**DOI:** 10.33549/physiolres.935545

**Published:** 2025-06-01

**Authors:** Xu ZHANG, Yunan KANG, Tongjun LI

**Affiliations:** 1 Second Affiliated Hospital of Heilongjiang University of Chinese Medicine, Heilongjiang University of Chinese Medicine, Harbin, Heilongjiang, China; 2 School of Basic Medical Sciences, School of Basic Medicine Sciences, Shandong Second Medical University, Weifang, Shandong, China

**Keywords:** Acupuncture, Electroacupuncture, Ulcerative colitis, Intestinal inflammation, Intestinal barrier

## Abstract

Ulcerative colitis (UC) is a chronic inflammatory bowel disorder. Currently, the global incidence of UC has significantly increased. Traditional treatment methods are relatively limited, with generally poor efficacy and many side effects. In contrast, acupuncture holds great promise due to its significant efficacy, reduced relapse rate, and minimal side effects. In recent years, basic research on acupuncture treatment for UC has achieved substantial progress. However, the specific targets and pathways involved are still unclear. Therefore, this review aims to summarize and consolidate the mechanisms of acupuncture and acupuncture combined with drug therapy for UC. We primarily review the mechanisms of acupuncture treatment for UC from two aspects: intestinal inflammation and intestinal barrier. In terms of intestinal inflammation, acupuncture improves UC by regulating various inflammatory molecules such as TNF-α, IL-1β, IL-10, NF-κB, and immune cells such as neutrophils, Th1, Th2, Treg, Th17. Concerning the intestinal barrier, we focus on the impact of acupuncture on the damage to intestinal epithelial cells (IECs). Moreover, acupuncture also possesses the capacity to reshape the gut microbiota, thereby repairing the biological barrier. Furthermore, the combination of acupuncture and medication for treating UC is a promising direction, which requires further exploration by researchers. This review thoroughly explains the molecular mechanisms of acupuncture in treating UC, establishing a foundation for further research on the effectiveness of acupuncture in UC treatment and offering a new perspective on the combination of acupuncture and medication.

## Introduction

Ulcerative colitis (UC) is a chronic idiopathic inflammatory disease that occurs in the mucosa of the colon [1,2]. UC is a multifactorial autoimmune disease that is thought to be the result of an interaction between genetic susceptibility, immune factors, and alterations in the intestinal microbiota, ultimately leading to an abnormal mucosal immune response and impaired epithelial barrier function. Dysregulation of the immune response in UC involves both the innate and adaptive immune systems. Although the mechanisms are unclear, immune cells such as T cells, B cells and macrophages, as well as several cytokines and chemokines that regulate the immune response, have been implicated in the pathogenesis of UC [3]. The intestinal flora also plays a crucial role in forming the intestinal barrier, enhancing the immune response of the intestinal mucosa and maintaining the intestinal environment. An imbalance in the intestinal flora can lead to intestinal barrier damage, further exacerbating the development of UC. The etiology of ulcerative colitis is still unclear, and it involves the complex interplay of genetic, environmental, and microbial factors. The primary clinical manifestations of UC include hematochezia, abdominal pain, and diarrhea [2]. Additionally, due to the persistent and chronic nature of this disease, UC patients are more likely to develop colorectal cancer than the general population [4,5]. In recent years, with the improvement of living standards and advancements in diagnostic technologies, the incidence of UC has significantly increased in both developed and developing countries, making it a global burden [1]. Consequently, clarifying the potential mechanisms of UC and determining more effective and stable treatment strategies has become an urgent task.

The main conventional treatments for UC are biologics and oral small molecule drugs (SMDs). SMDs entered clinical treatment as early as 1955, such as corticosteroids (e.g., budesonide, prednisone), immunomodulators (e.g., azathioprine, 6-mercaptopurine, and methotrexate), and aminosalicylates (e.g., sulfasalazine and mesalazine) [6,7]. SMDs have stable structures, and their production cost is relatively cheaper than that of biologics. Moreover, unlike biologics that require parenteral administration, oral SMDs can enhance patient satisfaction and improve treatment adherence and efficacy [8]. Biologics refer to a class of molecules, including recombinant cytokines, monoclonal antibodies, and specific antagonists of cytokines and soluble receptors, which regulate inflammation in immune-mediated processes. Among them, vedolizumab is the first anti-integrin approved for ulcerative colitis and is considered the safest biologic with the fewest side effects [9]. Infliximab, adalimumab, golimumab, and similar agents have shown significant efficacy in UC treatment [10]. Biologics exhibit higher immunogenicity compared to SMDs and are ideal for patients who suffer from severe side effects from other UC treatments, such as immunosuppressants or steroids [11]. Surgery would cause extensive loss of colon and rectum [12]. Although long-term medication can cause more severe disabilities than the surgical option, it is still considered an alternative treatment strategy and the last option in treatment plans [13,14].

One of the main challenges in the treatment and management of UC is that nearly all available UC therapies are associated with numerous adverse side effects [15,16]. As a result, interest in complementary and alternative medicine (CAM) for treating ulcerative colitis is increasing. According to reports, about 21–60 % of UC patients have utilized CAM, including acupuncture, herbal therapy, and mind-body interventions [17]. Acupuncture, as a potentially effective treatment for ulcerative colitis, offers excellent safety and minimal side effects. Therefore, acupuncture is increasingly viewed as a feasible adjunctive method to other management strategies and is widely applied in clinical settings [18–20]. An increasing number of studies revealed the effective mechanisms of acupuncture, and these studies have found that the initiation of the microenvironment at acupoints due to acupuncture stimulation is a central component. The acupoint microenvironment is rich in nerve endings, and the abundance of nerve receptors is the basis of the patient's needling sensation. The microenvironment of acupoints is also densely distributed with blood vessels, connective tissues, many types of cells (keratinocytes, mast cells, fibroblasts, macrophages), and different molecules (ATP, adenosine, HA, free ions, etc.), and the above components and their multidirectional interactions constitute the microenvironment of acupoints [21]. Acupuncture works by stimulating the microenvironment of acupuncture points, including the process of anatomical changes, regulation of cellular functions and release of various bioactive substances. Studies have shown that acupuncture promotes mast cell degranulation at acupoints, accompanied by electrical signaling and secretion of biochemicals such as trypsin-like enzymes, 5-hydroxytryptamine (5-HT) and histamine (HA), which have been shown to mediate the analgesic effects of acupuncture. However, through the release of these important chemicals, mast cells are closely associated with the effects of acupoint sensitization [22]. Acupoint sensitization is the transition of an acupoint from a physiological “resting state” to a pathological “active state”. Various sensitization phenomena occur after this transition, including sensations of soreness, swelling, itching, numbness, and pain. Mast cells in acupoint sensitization and acupuncture analgesia may respectively reflect their negative and positive effects. After the accumulation of changes in acupoint activity, acupuncture signals are transmitted to the central nervous system (CNS), leading to modulation of organoleptic function. As an example, in the most common pain disorder of acupuncture, during acupuncture analgesia, signals are transmitted from the acupoints to the CNS. The secretion of neurotransmitters and neuromodulators (e.g., opioid peptides, serotonin, norepinephrine, and dopamine) are then released from the spinal cord and the brain to play a combined role in suppressing pain [23].

**Fig. 1:**
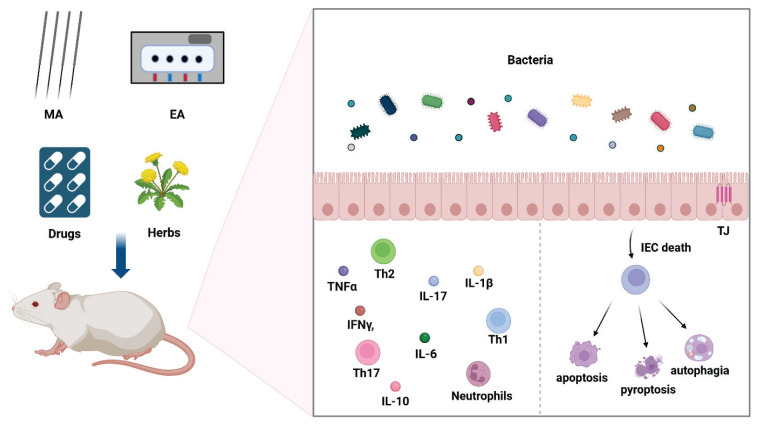
The mechanisms of acupuncture and acupuncture combined with drugs in the treatment of UC. The primary mechanisms of acupuncture in treating UC include inhibiting inflammatory molecules and cells and preventing the programmed death of IECs. The gut microbiota and TJ proteins are also crucial for the therapeutic effects of acupuncture.

However, the molecular mechanisms of acupuncture in UC patients have not been fully elucidated, and we still know very little about its targets and pathways. From the above, it is clear that intestinal inflammation and mucosal dysfunction play a crucial role in the pathogenesis of UC. Previous study has outlined the mechanisms through which acupuncture promotes the intestinal barrier, yet this research has neglected the important pathophysiological process of intestinal inflammation. This review mainly explains the molecular mechanisms of acupuncture on UC in a more comprehensive and systematic manner, focusing on intestinal inflammation and the mucosal barrier, and acupuncture combined with medication. It further explores the prospects and challenges of using acupuncture to treat UC. All of these provide a solid theoretical foundation for acupuncture therapy in UC treatment (Fig. 1).

## Intestinal inflammation

Immune response dysregulation is a significant factor in the pathogenesis of UC [2]. Modern experimental studies show that acupuncture offers significant benefits in modulating immune function in the body, aiding in the normalization of disrupted immune functions and improving disease resistance, mainly through the regulation of immune molecules and cells [21]. Immune molecules mainly include TNF-α, IL-1β, IL-12, IL-17, IL-4, IL-10, IL-6, NF-κB, while immune cells mainly include Neutrophil cells, Th1, Th2, Treg, and Th17.

IL-1β can enhance the expression of adhesion molecules on endothelial leukocytes, which induces a range of chronic intestinal inflammatory responses and tissue damage [24]. IL-12 and IL-17 are significant pro-inflammatory factors, with their expression elevated in the mucosa of active UC and their levels correlating with disease activity [25]. IL-4 is an anti-inflammatory cytokine that acts as a stimulator for B cells and T cells, exerting an immunosuppressive effect in the colon [26]. In addition, IL-10 also attenuates the inflammatory process in the mucosa, and a reduction in IL-10 levels was found in UC models [27]. Studies suggest that the mechanism of electroacupuncture at Shangjuxu (ST37) for UC in rats may involve the decrease of pro-inflammatory cytokine IL-1β and the increase of anti-inflammatory cytokine IL-4 [28]. Additionally, electroacupuncture using the acupoint formula “Chang Bing Fang” can downregulate the expression of pro-inflammatory nuclear factor kappaB p65 (NF-kappaB p65) protein in rats and upregulate serum anti-inflammatory cytokine IL-4 levels, which may help improve ulcerative colitis [29]. YanPing Chen's study aimed to further elucidate the mechanism. After applying electroacupuncture to the acupoints “Tianshu” (ST 25) and “Qihai” (CV 6), researchers found that acupuncture could modulate the balance between Th1 and Th2 cells in the colon tissue of UC rats, and improve immune function by downregulating INF-γ and IL-12 levels and upregulating IL-4 and IL-10 levels [30]. Notably, there may be differences in efficacy between electroacupuncture and manual acupuncture for UC. After intervening with electroacupuncture and manual acupuncture at “Quchi” (LI 11) and “Zusanli” (ST36) in the manual acupuncture group, pro-inflammatory factors TNF-α, IL-1β, and IL-6 were significantly downregulated, while the anti-inflammatory factor IL-10 was markedly further increased in the manual acupuncture group. Moreover, TNF-α is a key cytokine in UC pathogenesis, acting through the expression of adhesion molecules, pro-coagulants, and triggering apoptosis and acute-phase responses, and it also further enhances the production of IL-1β, IL-6, and IL-33 [26,31]. Electroacupuncture at Zusanli (ST36) can exert therapeutic effects on UC by downregulating serum TNF-α and colonic TNF-α mRNA [32]. Further research has found that electroacupuncture at ST36 improves colonic inflammation in rats by significantly increasing vagal nerve activity, which suppresses the levels of pro-inflammatory cytokines TNF-α, IL-1β, and IL-6 [33]. Additionally, Zusanli (ST36) is an effective acupoint for regulating gastrointestinal motility, protecting the gastric mucosa, and stimulating glandular secretion, and it can also enhance post-inflammatory rectal hypersensitivity and compliance, possibly *via* the NGF/TrkA/TRPV1 peripheral sensory pathway triggered by mast cells [34]. It is well known that NF-κB is also an important pro-inflammatory protein. Electroacupuncture at “Shangjuxu” (ST37) and “Tianshu” (ST25) can effectively mitigate colonic injury in UC rats, potentially by suppressing colonic NF-κB/Nod-like receptor family, pyrin domain-containing 3 (NLRP3) inflammasome signaling and reducing serum levels of IL-1β, TNF-α, and NLRP3 [35]. Qiao *et al*. further explored the upstream pathways of NF-κB and discovered that electroacupuncture might offer protective effects on the colonic mucosa of UC rats by inhibiting the Toll-like receptor 4/myeloid differentiation factor 88/nuclear factor-kappa B (TLR4/MyD88/NF-κB) signaling pathway, consequently reducing the release of inflammatory cytokines. EA intensity is another influencing factor, with high-intensity EA potentially providing better outcomes than low-intensity EA [36]. Chinese herbs, an important component of TCM, are also potential therapeutic agents for UC. For example, ginsenoside Rg1 (GS Rg1) ameliorates inflammation and oxidative stress in colitis through the Nrf-2/HO-1/NF-κB pathway [37].

**Fig. 2:**
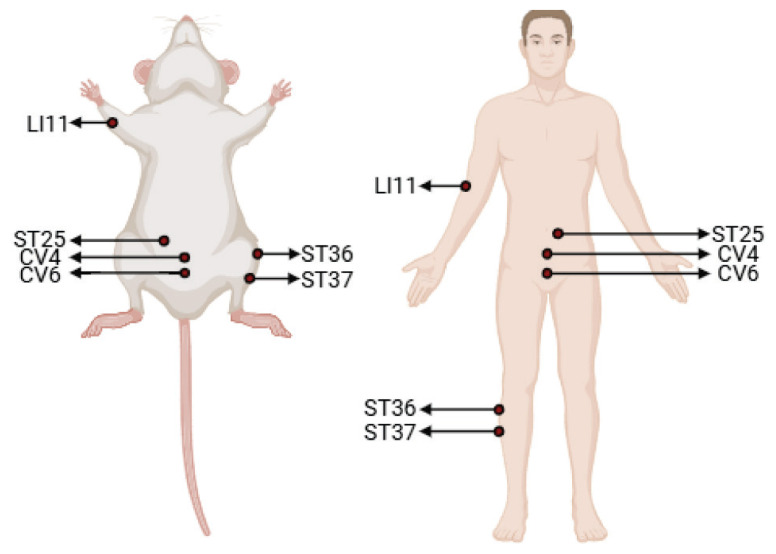
Diagram of acupuncture points in human and in the rat. The figure illustrates the acupuncture points commonly utilized for ulcerative colitis treatment in both clinical practice and rat experiments, all of which have been conclusively demonstrated to exert therapeutic effects against this condition.

One characteristic of UC is the presence of a large number of neutrophils in the intestinal lamina propria, whose persistent presence leads to tissue damage and the resulting chronic inflammatory cycle [38]. Hence, the regulation of neutrophil imbalance has emerged as a critical target in the treatment of UC. Studies have shown that acupuncture can improve the symptoms of UC rats by promoting neutrophil apoptosis and downregulating monocyte cytokines [39]. Moreover, the disturbed balance between regulatory T cells and effector T cells is another crucial pathogenic mechanism in UC [1,40]. Although previous studies indicated that Th1/Th2 might play a key role in the development of UC, recent research has found that the CD4 T cell subsets Treg and Th17 form a new immune axis closely associated with the onset and progression of UC [41]. Electroacupuncture at “Guanyuan” (CV 4) and “Zusanli” (ST36) can alleviate active-phase symptoms of UC mice by regulating the balance between the spleenic Treg and Th17 lymphocytes [42]. Another study also showed that electroacupuncture could improve the Treg/Th17 balance in UC mice [41] (Fig. 2).

Omics approaches, including genomics, proteomics, lipidomics, and metabolomics, have enabled comprehensive characterization of various molecular targets in UC, facilitating a deeper understanding of the disease mechanisms. Studies have demonstrated that electroacupuncture applied to the “Tianshu” (ST25) acupoint in UC rats significantly reduces colonic inflammation levels and damage, suggesting a protective role of electroacupuncture on the colon in UC rats. Transcriptomic sequencing by researchers revealed that differentially expressed genes in the electroacupuncture group were associated with the NF-κB signaling pathway, Toll-like receptor signaling pathway, PI3K-Akt signaling pathway, MAPK signaling pathway, and Wnt signaling pathway [43]. Previous studies have shown that the PI3K-Akt signaling pathway mediates the development of UC by promoting inflammation and activating T cells [44]. Circulating levels of Wnt1-inducible signaling pathway protein 1 (WISP-1) are increased in children with inflammatory bowel disease [45]. However, the critical target genes in acupuncture treatment for UC remain unclear. Zhang and colleagues, through transcriptomic sequencing, identified that the chemokine CXCL1 is a key target in the acupuncture treatment of UC, with its potential immune mechanism being related to the Th1 cytokine IFN-γ [46]. Studies have shown that chemokines play a crucial role in the pathogenesis of UC, primarily by recruiting neutrophils to the intestine and inducing various inflammatory responses, including neutrophil activation, degranulation, and the production of metalloproteinases to degrade the matrix [26]. Additionally, the proteome can be considered a hallmark of disease, as it is essentially the result of the interaction between genetic background and environmental factors [47]. Proteomics techniques have revealed various biomarkers related to UC immunity and inflammation, providing new insights and directions for studying the mechanisms of acupuncture in treating UC. For instance, proteomic analysis suggests that acupuncture may alleviate colonic inflammation in UC rats by regulating the expression of proteins related to the oxidative phosphorylation pathway, such as ATP synthase subunit g (ATP5L), ATP synthase beta subunit precursor (Atp5f), and cytochrome c oxidase subunit 4 isoform 1 (Cox4i1) [48]. Among these “omics” approaches, metabolomics remains the least applied and underutilized. Combining metabolomic analysis with *in vivo* anti-inflammatory activity studies in various animal models, such as colitis models, can provide deeper insights into disease mechanisms and identify potential therapeutic targets.

## Intestinal barrier

UC is characterized by its relapsing nature, which imposes a heavy disease burden and impairs quality of life. Recent evidence indicates that attaining clinical remission alone is insufficient for long-term remission. To achieve a favorable prognosis, mucosal healing (MH) has been defined as a therapeutic goal for UC [49]. The breakdown of the intestinal barrier and the loss of mucosa are fundamental aspects of UC. The intestinal barrier is divided into four parts: the mechanical barrier, which includes intestinal epithelial cells (IECs) and tight junctions (TJs); the biological barrier, mainly represented by the gut microbiota; the chemical barrier, primarily represented by the mucus layer and digestive enzymes; and the immune barrier, mainly represented by immune cells [50]. This review will focus on mechanical barriers and biological barriers.

## Mechanical barrier

The cellular structure of the intestine is marked by continuous IECs, which carry out essential innate immune functions such as antigen presentation, production of antimicrobial peptides and mucus, and maintenance of a tight physical barrier. IECs serve as the first line of defense against environmental and microbial attacks [51,52]. Different types of IECs ensure the division of labor and the efficient execution of various functions. For example, absorptive enterocytes absorb nutrients and present antigens [51]. Goblet cells help defend against invasive pathogens by secreting antimicrobial molecules such as trefoil factors and mucins [53]. Paneth cells contribute to innate host defense by secreting high levels of antimicrobial peptides in the intestinal crypts, with the induction of these peptides being closely related to the function of the intestinal barrier [54].

One of the strategies used by IECs to maintain homeostasis and intestinal barrier integrity is the critical ability to regulate the types of cell death. Eukaryotes have evolved the ability to regulate different modes of cell death, thereby facilitating various biological processes such as development, immunity, inflammation, and regeneration. Various molecular pathways, including apoptosis, necrosis, pyroptosis, and autophagy, eventually result in the death of IECs. Furthermore, tight junction (TJ) proteins, which are among the most essential proteins in the epithelial protective barrier, are vital in maintaining the integrity of the intestinal epithelium [55]. Damaged TJ barriers increase paracellular permeability and trigger a series of events, including cell apoptosis, which results in intestinal epithelial damage [56]. Therefore, the imbalance of IECs homeostasis and the dysfunction of TJs are considered key causes of the mechanical barrier dysfunction of the intestinal mucosa.

Cell apoptosis is determined by the relative expression of sequential genes that regulate the process. The Fas/FasL pathway plays a crucial role in epithelial cell apoptosis in UC [57,58]. The apoptosis-promoting gene Bax also plays a crucial role in the process of apoptosis. The ratio of bax to bcl-2 dictates the occurrence of apoptosis, with an overexpression of bax promoting apoptosis [59]. Studies have found that acupuncture can regulate the expression of Bcl-2, Bax, Fas, and FasL proteins in rats through the Bcl-2/Bax and Fas/FasL pathways, thereby inhibiting epithelial cell apoptosis in ulcerative colitis [60]. Additionally, Huangan Wu *et al*. found that acupuncture stimulation at “Qihai” (CV 6) and “Tianshu” (ST25) can significantly reduce abnormal epithelial cell apoptosis [61]. Besides electroacupuncture, manual acupuncture also has similar effects; both EA and manual acupuncture groups significantly inhibited the downregulation of the anti-apoptotic protein Bcl-2/Bax expression in the colon of UC rats [62]. The NLRP3 inflammasome serves as a critical target in pyroptosis. Electroacupuncture with the “intestinal disease prescription” has a significant therapeutic effect on DSS-induced UC, possibly by regulating the expression of the NLRP3 inflammasome and proteins related to the intestinal mucosal barrier, thereby alleviating UC symptoms [63]. Autophagy is an evolutionarily conserved process, primarily responsible for degrading endogenous biomolecules for recycling purposes [64]. Autophagy is closely related to the cellular stress response; in conditions of nutrient deprivation, autophagy is rapidly induced through self-digestion to maintain cell viability, and under nutrient-rich conditions, it promotes anabolic functions [65]. Numerous studies have shown that autophagy defects are closely related to the pathogenesis of UC, making autophagy inhibition a novel therapeutic strategy for treating UC [66]. Moreover, autophagy is negatively regulated by the upstream protein kinase mTOR, both of which are central regulators of organismal growth and homeostasis [67]. Wu *et al*. found that EA of acupoint recipe “Changbingfang” can improve symptoms in UC rats, and its mechanism is related to promoting colonic autophagy, increasing AMPK phosphorylation levels, and reducing mTOR phosphorylation levels [68]. Moreover, autophagy induced by endoplasmic reticulum stress (ERS) plays a critical role in the pathogenesis of UC. ERS is a disruption of internal homeostasis triggered by the accumulation of misfolded or unfolded proteins in the endoplasmic reticulum (ER) [69]. ERS can induce autophagy via multiple pathways, including the Akt signaling pathway, the ATF4 signaling pathway, and the IRE1 signaling pathway [70,71]. Notably, both EA and MA treatments can significantly alleviate colonic inflammation in UC rats by inhibiting oxidative stress and ERS [72]. This indicates that inhibiting autophagy is also a critical target in acupuncture treatment for UC.

## Biological barrier

The gut microbiota is a vital part of the human microecosystem, contributing to important physiological processes like nutrient absorption and the development of the intestinal immune system, with its alterations being closely linked to the progression of numerous diseases [73]. An increasing number of studies suggest that compared to healthy adults, the total bacterial count in the UC gut microbiota is lower, with reduced diversity and altered metabolic activity [56]. Therefore, reshaping the gut microbiota has become an attractive therapeutic approach for UC patients. Acupuncture is a critical therapeutic method in Traditional Chinese Medicine (TCM) that can effectively balance the gut microbiota, enhancing the abundance of beneficial bacteria while reducing the abundance of pathogenic bacteria. After establishing the UC rat model, the researchers found that EA stimulation at “Tianshu” (ST25), “Zusanli” (ST36), and “Shangjuxu” (ST37) significantly reduced the Disease Activity Index (DAI) score and the content of enteric Clostridium bifermentans, while significantly increasing the abundance and diversity of bacterial populations, as well as the content of enteric Lachnospiraceae bacterium and Lactobacillus sp. in the gut, which may be related to its protective effect on the UC gut microbiota [74]. More importantly, gut microbiota imbalance can also damage intestinal barrier function, leading to increased intestinal permeability and inflammation. Adiponectin increases the expression of intestinal tight junction proteins and enhances intestinal barrier integrity. In a dextran sulfate sodium (DSS)-induced colitis mouse model, EA stimulation of ST36 not only regulates the gut microbiota but also promotes the recovery of adiponectin and alleviates systemic inflammation levels [75]. Another study indicates that the DSS-induced colitis group showed an increase in the abundance of Escherichia-Shigella and Clostridium perfringens, while both high-frequency and low-frequency electroacupuncture treatments activated the mitogen-activated protein kinase (MAPK) signaling pathway, thereby maintaining the integrity of the intestinal barrier [76]. Additionally, numerous studies have shown that herbs and TCM formulas can modulate the composition of the gut microbiota. For instance, triptolide, Red Ginseng, and Semen Coicis have been shown to enhance the structure of the gut microbiota and alleviate symptoms of ulcerative colitis *in vivo* [77]. These studies indicate that the gut microbiota is a new target for acupuncture and even TCM in the treatment of UC.

Notably, fecal microbiota transplantation (FMT) and oral probiotics are becoming promising approaches to relieve active ulcerative colitis. FMT aims to correct dysbiosis by administering feces collected from a donor and has been used to treat many gut microbiome-related diseases, including UC [78]. However, Modern medicine still requires further exploration in areas such as healthy donor screening, fecal microbiota transplantation solution preparation, analysis of the effective components in the microbiota solution, and the mechanisms of FMT treatment. In this regard, records of FMT in TCM can be traced back to the 3^rd^ century AD, with related theories documented in many TCM texts throughout history. Fu *et al*. simulated the preparation environment for Chinese medicine “Jin Zhi”, anaerobically fermenting the prepared fecal microbiota suspension at 10 °C and 20 °C. They then gavaged colitis mice with both fresh fecal microbiota suspension prepared using this method and the suspension fermented for 10 days, and observed the alleviation of intestinal inflammation, along with the disease activity index, changes in gut microbiota composition, and colon histopathology. The research found that the number of anaerobic bacteria was higher and their activity was better in the simulated preparation of “Jin Zhi”, which also demonstrated enhanced antibacterial capability [79]. Therefore, it is evident that TCM offers a unique perspective on FMT transplantation. Furthermore, research indicates that probiotics may contribute to the treatment of UC, with key mechanisms including maintaining normal gut microbiota balance, enhancing intestinal mucosal barrier function, promoting immune tolerance in the intestinal mucosa, interfering with intestinal inflammatory responses, and inhibiting apoptosis of intestinal epithelial cells [80]. A meta-analysis shows that Lactobacillus and Escherichia coli can significantly improve UC. Kimchi, a plant-based probiotic food fermented mainly by Lactobacillus, is believed to have an effect on UC, though its role still requires further research [81].

## Acupuncture combined with medication

UC is a chronic relapsing disease that requires long-term medication. However, commonly used UC drugs almost always have adverse effects. Therefore, using acupuncture combined with medication to enhance drug dependence and tolerance in UC patients has become a treatment option in urgent need of development. Studies have already provided new evidence supporting the efficacy of acupuncture either combined with or as an alternative to drug therapy for UC [19]. The combination of Ge Xia Zhu Yu Decoction and acupuncture at Zusanli (ST36) can effectively enhance the therapeutic effect in patients with active UC, decrease relapse rates, alleviate clinical symptoms, and quickly restore the mucosa [82].

Compared to traditional UC drugs, the active ingredients of Chinese herbal medicine and herbal formulas feature multiple regulatory targets, stable structures, and high safety, offering significant benefits in UC treatment and emerging as a new focus in current research. Growing evidence indicates that several herbs, including aloe vera gel, curcumin, natural indigo, and Andrographis paniculata extract, can improve clinical symptoms and reduce relapse rates [83,84]. A randomized clinical trial discovered that HMPL-004, an extract of Andrographis paniculata, may serve as an effective alternative to mesalamine for treating ulcerative colitis [85]. Fu Fang Ku Shen Colon Capsule (FCC) is a Chinese herbal medicine used for treating UC. A randomized double-blind study evaluated the therapeutic potential of FCC in patients with active UC. Out of 320 patients with active UC, they were assigned to receive either FCC or mesalamine for a duration of 8 weeks. At the end of the 8 weeks, 72.5 % of patients in the FCC group and 65 % in the mesalamine group achieved clinical remission [86]. Qing Chang Hua Shi (QCHS) is a Chinese herbal formula used for UC patients at the Affiliated Hospital of Nanjing University of Chinese Medicine. Our study evaluating the clinical effects of QCHS has shown encouraging results. Sixty UC patients were randomly assigned to either the QCHS group or the mesalamine group for 8 weeks. Endoscopic scores improved in both groups. No significant differences were observed between the QCHS group and the mesalamine group regarding mucosal healing and clinical remission [87].

## Discussion

UC continues to result in high incidence rates and substantial productivity losses globally. Additionally, the recurring nature of UC necessitates repeated treatments, which imposes a significant economic burden on individual patients and healthcare systems, especially in developing countries. With the introduction of biologics such as anti-TNF drugs, the rate of early surgery in UC patients has decreased. Nonetheless, existing UC treatments continue to face challenges, highlighting the urgent need for new therapeutic options. Acupuncture is a crucial treatment in TCM, widely embraced by healthcare professionals and patients for its notable therapeutic benefits, safety, and environmental sustainability. Therefore, acupuncture, being a non-invasive treatment with minimal adverse side effects, holds great potential for further development. However, the mechanism by which acupuncture treats UC remains unclear and needs further investigation. This article mainly reviews the molecular mechanisms of acupuncture in treating UC from the aspects of intestinal inflammation and intestinal barrier, as well as the latest progress in acupuncture combined with drug treatment for UC, providing new strategies for UC treatment.

From the above content, we learn that maintaining the integrity of IECs is a key method for treating UC, and acupuncture helps suppress the programmed death of IECs, offering protection to the intestinal mucosal barrier. Programmed cell death (PCD) is a mechanism of cell death involving complex signaling pathways that occur in a regulated and orderly manner, controlled by genes. It primarily encompasses apoptosis, autophagy, ferroptosis, pyroptosis, and necroptosis. Apoptosis and autophagy are established as primary forms of PCD and play crucial roles in the treatment of UC. Additionally, numerous studies indicate a close association between ferroptosis and UC. It differs from apoptosis, necroptosis, and autophagy in terms of morphology, biochemistry, and genetics, and is classified as a non-apoptotic form of cell death [88]. Ferroptosis is attributed to the accumulation of excessive lipid reactive oxygen species (ROS), leading to the peroxidation of polyunsaturated fatty acids (PUFAs) and membrane damage [89]. Studies have shown that targeting ferroptosis represents a new approach in TCM for treating ulcerative colitis. This research mainly investigates how effective components and formulas of TCM improve UC by inhibiting ferroptosis [90]. Nevertheless, as an important component of TCM, research on the mechanisms of ferroptosis in UC related to acupuncture is still in the exploratory stage, and many issues remain to be addressed in the future. Additionally, during the process of maintaining homeostasis, damage to the intestinal epithelium triggers a regenerative response. The phagocytosis of dead cells and mediators derived from these cells (such as prostaglandin E2 (PGE2)) or microvesicle components derived from apoptotic cells (such as CRKL) leads to the resolution of inflammation and the initiation of compensatory proliferation, thereby replenishing lost epithelial cells and restoring homeostasis [91,92]. To date, it is still unknown whether acupuncture plays a role in the regenerative response of IECs.

Interestingly, intestinal barrier dysfunction can be detected in asymptomatic relatives of patients with inflammatory bowel disease even before the development of intestinal pathology [93]. This indicates that acupuncture could be a significant avenue for future prevention and treatment of UC. Meanwhile, the potential regulatory effects of other traditional Chinese medical therapies, such as moxibustion, tui na, and Chinese herbal medicine, also require further research [94]. Most studies on the active components of Chinese herbal medicine and herbal formula components are based on animal experiments and *in vitro* cell studies, lacking clinical evidence. Furthermore, the quantitative relationship between the concentration of effective drugs in the human body and the efficacy of TCM warrants further investigation.

In conclusion, researchers in acupuncture should persist in detailed exploration and summarization of UC-related signaling pathways and molecular targets in future studies, and employ advanced omics techniques to further screen the expression of relevant genes in UC.
